# Comet and cytogenetic tests as tools for evaluating genomic instability in seeds of *Oryza sativa* L. and *Phaseolus vulgaris* L. from gene banks

**DOI:** 10.1590/1678-4685-GMB-2017-0020

**Published:** 2018

**Authors:** Alisson F. Dantas, Renata M. Lopes, Maria L. Fascineli, Solange C.B.R. José, Juliano G. Pádua, Marcos A. Gimenes, Cesar K. Grisolia

**Affiliations:** 1 Laboratório de Genética Toxicológica, Departamento de Genética e Morfologia, Instituto de Ciências Biológicas, Universidade de Brasília, Brasília, DF, Brazil; 2 Departmento de Botânica, Instituto de Ciências Biológicas, Universidade de Brasília, Brasília, DF, Brazil; 3 Embrapa Recursos Genéticos e Biotecnologia, Brasília, DF, Brazil

**Keywords:** *Oryza sativa* L., *Phaseolus vulgaris* L., methyl methanesulfonate, cytogenetic test, comet test

## Abstract

This study aimed to assess the feasibility of comet and cytogenetic tests as tools for evaluating genomic instability in seeds of *Oryza sativa* L. (rice) and *Phaseolus vulgaris* (beans) L. from gene banks. Rice and beans were exposed to methyl methanesulfonate (MMS) as a reference DNA damaging agent. Seeds of two accessions of rice and beans were obtained from Embrapa Rice and Beans - Brazil. Seed groups were imbibed in three concentrations of MMS for three periods of time to carry out cytogenetic tests, and for one period for the comet test. At concentrations of 10 and 15 mg/L, MMS induced cytotoxic and/or mutagenic effects in the meristematic cells of roots from all the accessions of both species. In the comet test, MMS induced genotoxic effects at all the concentrations in the evaluated accessions of rice and beans, except in one accession of beans at the lowest concentration (5 mg/L). Both species showed sensitivity to MMS. The comet test can be proposed for the measurement of genomic instability in accessions of rice and beans in gene banks, as being more sensitive than the cytogenetic tests used.

## Introduction

Common rice, *Oryza sativa* L. (Poaceae family), and common bean, *Phaseolus vulgaris* L. (Fabaceae family) play an important role in the nutrition of various countries, and in Brazil they are the main components of the staple diet (Barbosa, 2007). Common rice is an annual gramineous species of Asiatic origin, which can adapt to a wide range of environmental conditions (Sousa *et al.*, 1995), and it is widely distributed in tropical and subtropical regions and in some temperate regions (Vaughan, 1994). Common bean is a leguminous species that presents a large number of varieties and cultivars spread over different continents, due to the high value of its seeds for human nutrition (Peixoto *et al.*, 2000). Consumption of both can benefit human health due to the presence of antioxidant compounds (Walter and Marchesan, 2011; López *et al.*, 2013).

Because of the importance of rice and beans as food items, large germplasm collections have been established, and there are studies reporting cytotoxic, mutagenic and/or genotoxic evaluations in *O. sativa* (Mei *et al.*, 1994; Todoriki and Toru, 1999; Mohanty *et al.*, 2004; Wei *et al.*, 2006; Shi *et al.*, 2010; Endo *et al.*, 2012; Kwon *et al.*, 2013; Macovei and Tuteja, 2013; Macovei *et al.*, 2014) and in *P. vulgaris* (Hallak *et al.*, 1999; Khan *et al.*, 2002, Cenkci *et al.*, 2010). The use of methodologies to detect damage to DNA based on chromosome breaks and mis-segregation would play an important role in understanding genomic stability and the viability of seeds stored in gene banks, because the loss of genomic stability is one of the first stages in the seed deterioration process (Peixoto *et al.*, 2006). In this case, methyl methanesulfonate (MMS) was used as reference compound for inducing such genomic damages in rice and beans. MMS is an alkylating agent capable of adding methyl groups to a series of nucleophilic sites in DNA bases (Wyatt and Pittman, 2006; Malini *et al.*, 2010), which can cause chromosome aberrations (Kaina, 2004). It is used for mutagenic and genotoxic evaluation in different organisms, from fungi (Myung and Kolodner, 2003) to rodents (Muto *et al.*, 2015; Plappert-Helbig *et al.*, 2015).

The main goal of the present study was to test known cytogenetic methodologies and single cell gel electrophoresis (comet test), as tools for the measurement of genomic instability in seeds stored in a gene bank.

## Material and Methods

### Plant material

Seeds from two accessions of *O. sativa* (BGA012099 “Ferrinho” and BGA008070 “Primavera”) and from two accessions of *P. vulgaris* (GF004 and GF007) were obtained from the Active Germplasm Bank of Rice and Beans (Embrapa Rice and Beans, Santo Antônio de Goiás, GO, Brazil). They were stored for six months at 10 °C and 30% of humidity. Initial germination of the seeds was 97 and 94% for the accessions of *O. sativa*, BGA012099 “Ferrinho” and BGA008070 “Primavera”, respectively, and 56 and 99% for the accessions of *P. vulgaris*, GF004 and GF007, respectively.

### Chemicals

MMS (CAS N. 66-27-3), ethidium bromide, sodium dodecyl sulfate, tris (hydroxymethyl) methane, ethylene diamine tetraacetic acid (EDTA) and boric acid were acquired from Sigma-Aldrich (St. Louis, USA). Normal-melting-point (NMP) agarose was acquired from Life Technologies (New York, USA) and low-melting-point (LMP) agarose from Laboratorios Conda (Madrid, Spain).

### Cytogenetic tests

Twenty seeds from two accessions of *O. sativa* (2*n* = 2*x* = 24) and 30 seeds from two accessions of *P. vulgaris* (2*n* = 2*x* = 22) were imbibed in different concentrations of MMS for three periods of time: MMS 5 mg/L for 4, 8 and 24 h; MMS 10 mg/L for 4, 8 and 24 h and MMS 15 mg/L for 4, 8 and 24 h. As well as these treatments, seeds from the different accessions were imbibed in only distilled water for 24 hours. As a control, seeds of *O. sativa* and *P. vulgaris* were used without exposure to MMS or distilled water. Next, the seeds from each treatment were sown in substrate of germitest paper, wetted with distilled water, at the proportion of 2.5 mL/g of dry paper. The germitest papers with seeds remained at room temperature (20-30 °C) until the roots reached between 1 and 2 cm for *O. sativa*, and between 1 and 3 cm for *P. vulgaris*. The roots were fixed in Carnoy solution (ethanol/glacial acetic acid - 3:1 v/v) and stored in ethanol 70% until slides were prepared.

The slides were prepared according to the methodology proposed by Guerra and de Souza (2002), with modifications. The roots were washed in distilled water for 5 min, hydrolyzed in hydrochloric acid (5N) for 25 min, washed again in distilled water for 2 min and arranged on slides to cut the meristematic region of the roots and then to color with acetic orcein 2% for 15 min. Next, the meristematic region was covered with a coverslip and evaluated under an optical microscope (1,000 X).

Evaluation of 1,000 cells per root was carried out, with four roots per group, to a total of 4,000 cells analyzed per group tested. The parameters used to characterize the groups were: (a) Mitotic Index (MI) as an indication of cytotoxicity, (b) frequency of chromosome aberrations, and (c) frequency of micronuclei (MNs) as an indication of mutagenicity. For the analysis of chromosome alterations, we evaluated chromosome lagging and chromosome bridges in anaphase and/or telophase and chromosome fragments in metaphase, anaphase and/or telophase. The evaluation of the slides was carried out in a blind test.

### Comet test

Ten seeds from the two accessions of *O. sativa* and from the two accessions of *P. vulgaris* were imbibed in different concentrations of MMS for a single period of time: MMS 5 mg/L for 24 h; MMS 10 mg/L for 24 h and MMS 15 mg/L for 24 h. Similar to the cytogenetic tests, one group of seeds of *O. sativa* and *P. vulgaris* was also submitted to imbibition in distilled water for 24 h. The control consisted of seeds of *O. sativa* and *P. vulgaris* without exposure to MMS or distilled water.

Cell suspensions were obtained from seed embryos from the two accessions of rice and bean for consequent processing in the test. Cell suspensions were obtained in accordance with Koppen and Cerda (1997), with some adaptations. The embryos collected were transferred to 2 mL microtubes containing 1 mL of cold phosphate buffered saline (PBS), macerated and left for 1 h in the refrigerator. Next, the supernatant was used to carry out the comet test.

The comet test was carried out in accordance with Cerda *et al.* (1997), with modifications. Fifteen microliters of the cell suspension were mixed with 85 μL of LMP agarose (0.8%) at 45 °C and arranged on slides pre-covered with NMP agarose (0.5%). Next, the slides were re-covered immediately with the coverslip, placed on a metal sheet, and put in the refrigerator for 5 min. After the agarose gel solidified, the coverslips were removed and the slides immersed in TBE buffer solution (45 mM Tris-borate, 1 mM EDTA, pH 8.4) containing 2.5% sodium dodecyl sulfate for 30 min. After the lysis phase, the slides were transferred to an electrophoresis tank containing TBE buffer solution and left to rest for 5 min before running electrophoresis at 0.5 V/cm for 2 min. To determine the most appropriate voltage for the observation of nucleoids of *O. sativa* and *P. vulgaris* embryos, SCGE tests were done with different voltages (0.5, 0.75, 1 and 2 V/cm for rice and 0.5, 0.75 and 1 V/cm for bean) for 2 min. After electrophoresis, the slides were plunged into ice-cold distilled water for 10 min, dried at room temperature and kept in the refrigerator until the moment for staining. The slides were stained with 50 μL of ethidium bromide (20 μg/mL) and analyzed under a fluorescence microscope (400 X) using excitation and emission filters of 546 nm and 590 nm. All the steps took place in weak or yellow light. Three slides were prepared for each group of both species, and on the slides of *O. sativa*, between 20 and 50 nucleoids were analyzed per slide, due to the small quantity of nucleoids obtained in some slides; on the slides of *P. vulgaris*, 50 nucleoids were analyzed per slide. The evaluation of nucleoids of *O. sativa* and *P. vulgaris* embryos was done using the *Comet Assay* IV program, version 4.3.1, and the parameter of tail intensity was chosen to measure the damage to DNA as being indicative of genotoxicity. Slides were evaluated in a blind test.

### Statistical analysis

The statistical analyses of the data from the cytogenetic and comet tests were done with GraphPad Prism software, version 5.00, using One-way ANOVA followed by the Tukey test with significance levels of *p*<0.05.

## Results and Discussion

Initially, seeds of *O. sativa* and *P. vulgaris* were exposed only to distilled water for 24 h to verify the influence of imbibition, as the excess of water could be damaging for the seeds and interfere indirectly in the oxygenation of the embryo (Black *et al.*, 2006). However, it was observed that imbibition of the seeds in water for 24 h did not induce observable damage (*p* ≥ 0.05) in the cytogenetic and comet tests in seeds from the two accessions of *O. sativa* and *P. vulgaris* that were evaluated.

### Cytogenetic tests

In the *O. sativa* accessions evaluated, MMS significantly inhibited the MI of meristematic cells of the roots of accession BGA012099 “Ferrinho” at the concentration of 15 mg/L for 4 and 8 h, when compared with the control (Table 1). In addition, MMS significantly reduced the MI of meristematic root cells of accession BGA008070 “Primavera” at the concentration of 15 mg/L for 4 h, when compared with the control (Table 2). In accession GF004 of *P. vulgaris*, MMS at the concentration of 10 and 15 mg/L for 24 h reduced the MI of the meristematic root cells of accession GF004, differing statistically from the other groups tested (Table 3). This indicated that exposure time may be a determining factor for the observation of these effects. In accession GF007, MMS significantly reduced the MI of the meristematic root cells at the concentration of 15 mg/L for 24 h, when compared to the control and to distilled water (Table 4).

**Table 1:** Mitotic index, frequency of chromosome aberrations and of micronuclei obtained in the cytogenetic tests on cells from the meristematic region (n = 4000) in roots of *Oryza sativa* L. accession BGA012099 “Ferrinho”, exposed to methyl methanesulfonate (MMS).

Groups	Mitotic Index	Chromosome aberrations				Micronuclei frequency
		Chromosome lagging	Fragments	Bridges	Total frequency	
Control	8.42 ± 0.91	0.25 ± 0.50	-	-	0.25 ± 0.50	-
Distilled water	6.82 ± 0.68	1.00 ± 0.81	0.25 ± 0.50	-	1.25 ± 0.50	-
**MMS 5 mg/L**						
4 h	7.42 ± 1.47	0.25 ± 0.50	-	-	0.25 ± 0.50	-
8 h	8.35 ± 0.42	1.00 ± 0.81	-	-	1.00 ± 0.81	-
24 h	7.77 ± 1.07	0.75 ± 0.95	-	0.50 ± 0.74	1.25 ± 0.95	-
**MMS 10 mg/L**						
4 h	7.67 ± 1.17	1.75 ± 0.95	0.75 ± 0.95	-	2.50 ± 1.00	0.25 ± 0.50
8 h	7.40 ± 0.60	1.25 ± 0.95	0.75 ± 0.50	0.75 ± 0.95	2.50 ± 1.29	0.25 ± 0.50
24 h	8.40 ± 0.49	1.75 ± 1.70	0.25 ± 0.50	0.75 ± 1.50	2.75 ± 1.50^a*^	-
**MMS 15 mg/L**						
4 h	6.27 ± 0.76^a*^	0.75 ± 1.50	0.50 ± 0.57	-	1.25 ± 1.25	-
8 h	6.02 ± 0.91^a**^	-	0.25 ± 0.50	0.50 ± 1.00	0.75 ± 0.95	-
24 h	6.95 ± 0.62	1.50 ± 1.73	-	-	1.50 ± 1.73	-

Control = seeds not exposed to MMS (methyl methanesulfonate) or to distilled water. (-) = not observed. The data are represented as means ± SD; ^a^ significant in relation to control; One-way ANOVA followed by Tukey test. (^*^p<0.05; ^**^p<0.01).

**Table 2:** Mitotic index, frequency of chromosome aberrations and of micronuclei obtained in the cytogenetic tests on cells from the meristematic region (n = 4000) in roots of *Oryza sativa* L. accession BGA008070 “Primavera” exposed to methyl methanesulfonate (MMS).

Groups	Mitotic Index	Chromosome aberrations				Micronuclei frequency
		Chromosome lagging	Fragments	Bridges	Total frequency	
Control	8.05 ± 1.30	-	-	-	-	-
Distilled water	6.77 ± 0.49	0.50 ± 0.74	-	-	0.50 ± 0.74	-
**MMS 5 mg/L**						
4 h	7.42 ± 1.47	0.50 ± 0.74	-	-	0.50 ± 0.74	-
8 h	8.35 ± 0.42	0.50 ± 1.00	0.50 ± 0.74	0.50 ± 1.00	1.50 ± 1.73	-
24 h	7.77 ± 1.07	-	0.50 ± 0.74	-	0.50 ± 0.74	-
**MMS 10 mg/L**						
4 h	7.40 ± 0.77	0.50 ± 1.00	1.25 ± 1.50	-	1.75 ± 1.25	0.25 ± 0.50
8 h	7.50 ± 0.50	1.00 ± 0.81	1.25 ± 0.95	0.50 ± 1.00	2.75 ± 1.50^a*^	0.50 ± 0.57
24 h	7.17 ± 2.14	0.75 ± 1.50	2.25 ± 1.25^a*;b*^	-	3.00 ± 1.15^a**;b*^	-
**MMS 15 mg/L**						
4 h	5.77 ± 0.85^a*^	0.75 ± 1.50	0.50 ± 0.57	0.25 ± 0.50	1.50 ± 2.38	-
8 h	7.00 ± 0.98	0.25 ± 0.50	0.25 ± 0.50	-	0.50 ± 0.57	-
24 h	7.85 ± 1.05	0.75 ± 0.95	0.75 ± 0.50	-	1.50 ± 1.29	-

Control = seeds not exposed to MMS (methyl methanesulfonate) or to distilled water. (-) = not observed. The data are represented as means ± SD; ^a^ significant in relation to control; ^b^ significant in relation to the distilled water group; One-way ANOVA followed by Tukey test. (^*^p<0.05; ^**^p<0.01).

**Table 3:** Mitotic index, frequency of chromosome aberrations and of micronuclei obtained in the cytogenetic tests on cells from the meristematic region (n = 4000) in roots of *Phaseolus vulgaris* L. accession GF004, exposed to methyl methanesulfonate (MMS).

Groups	Mitotic Index	Chromosome aberrations				Micronuclei frequency
		Chromosome lagging	Fragments	Bridges	Total frequency	
Control	5.92 ± 1.29	-	-	-	-	-
Distilled water	5.15 ± 1.00	-	-	-	-	-
**MMS 5 mg/L**						
4 h	5.67 ± 1.43	1.00 ± 1.15	0.75 ± 0.95	-	1.75 ± 1.70	-
8 h	5.17 ± 1.55	0.25 ± 0.50	0.50 ± 0.57	-	0.75 ± 0.95	-
24 h	3.95 ± 0.91	0.25 ± 0.50	0.75 ± 0.95	-	1.00 ± 1.15	0.50 ± 0.57
**MMS 10 mg/L**						
4 h	4.72 ± 0.49	0.25 ± 0.50	0.50 ± 0.57	-	0.75 ± 0.50	-
8 h	4.85 ± 0.68	1.00 ± 0.00^a**;b**;c*^	0.25 ± 0.50	-	1.25 ± 0.50^a*;b*^	-
24 h	2.72 ± 0.47^a***;b**;c*;d*^	0.50 ± 0.57	-	0.25 ± 0.50	0.75 ± 0.95	0.25 ± 0.50
**MMS 15 mg/L**						
4 h	7.87 ± 2.45	1.25 ± 0.50^a***;b***;c**;e***^	-	-	1.25 ± 0.50^a***;b***;c**;e***^	-
8 h	4.15 ± 0.56^c*^	0.25 ± 0.50	-	-	0.25 ± 0.50	-
24 h	1.82 ± 0.90^a**;b*;c***^	-	-	-	-	-

Control = seeds not exposed to MMS (methyl methanesulfonate) or to distilled water. (-) = not observed. The data are represented as means ± SD; ^a^ significant in relation to control; ^b^ significant in relation to the distilled water group; ^c^ significant in relation to the time of 4 h; ^d^ significant in relation to the time of 8 h; ^e^ significant in relation to the time of 24 h. One-way ANOVA followed by Tukey test. (^*^p<0.05; ^**^p<0.01; ^***^p<0.001).

**Table 4:** Mitotic index, frequency of chromosome aberrations and of micronuclei obtained in the cytogenetic tests on cells from the meristematic region (n = 4000) in roots of *Phaseolus vulgaris* L. accession GF007, exposed to methyl methanesulfonate (MMS).

Groups	Mitotic Index	Chromosome aberrations				Micronuclei frequency
		Chromosome lagging	Fragments	Bridges	Total frequency	
Control	7.90 ± 1.60	-	-	-	-	-
Distilled water	5.97 ± 1.21	0.25 ± 0.50	-	-	0.25 ± 0.50	-
**MMS 5 mg/L**						
4 h	8.00 ± 1.60	0.25 ± 0.50	-	0.25 ± 0.50	0.50 ± 0.57	0.25 ± 0.50
8 h	5.47 ± 2.10	0.25 ± 0.50	0.25 ± 0.50	-	0.50 ± 1.00	-
24 h	6.37 ± 2.65	0.25 ± 0.50	-	-	0.25 ± 0.50	-
**MMS 10 mg/L**						
4 h	8.15 ± 3.37	-	-	0.25 ± 0.50	0.25 ± 0.50	-
8 h	6.67 ± 1.21	0.25 ± 0.50	-	0.25 ± 0.50	-	-
24 h	5.17 ± 1.42	1.50 ± 1.73	0.25 ± 0.50	-	1.75 ± 1.70	-
**MMS 15 mg/L**						
4 h	8.35 ± 1.27	0.50 ± 1.00	0.25 ± 0.50	0.25 ± 0.50	1.00 ± 1.41	-
8 h	7.07 ± 1.87	0.50 ± 0.57	-	0.75 ± 1.50	1.25 ± 1.89	-
24 h	4.52 ± 0.95^a*;b*^	1.25 ± 1.25	0.25 ± 0.50	-	1.50 ± 1.29	-

Control = seeds not exposed to MMS (methyl methanesulfonate) or to distilled water. (-) = not observed. The data are represented as means ± SD; ^a^ significant in relation to the control; ^b^ significant in relation to the distilled water group. One-way ANOVA followed by Tukey test. (^*^p<0.05).

In mammals, MMS induces a collapse in the replication forks or a halt in replication due to the addition of methyl groups to the DNA molecule, preventing the progression of DNA replication in the cell cycle (Wyatt and Pittman, 2006). Although cytotoxic damages of MMS have not been previously reported in the literature with the plants evaluated in the present study, the cytotoxicity of MMS has been reported in meristematic cells of plant roots, such as *Allium cepa* L. (Rank and Nielsen, 1993), and it is well established as a positive control in *A. cepa* test (Liman *et al.*, 2015).

A series of events occur in the seed up until its complete germination. Seed metabolism begins after hydration. Respiration and synthesis of proteins begin minutes after hydration, followed by RNA synthesis, repair mechanisms and DNA synthesis. The last event in germination is the expansion of the cells in the radicle, preceding cell division. Most seeds do not manage to germinate when immersed in water, but the seeds of some aquatic plants, such as rice, do manage to germinate successfully in this condition (Black *et al.*, 2006). This characteristic of rice may help to explain the fact that no cytotoxic effects were observed occasioned by MMS in accessions of *O. sativa* in the period of 24 h for accession BGA012099 “Ferrinho”, and in the periods of 8 and 24 h for accession BGA008070 “Primavera”. The absorption of MMS in the first periods of exposure of these accessions of *O. sativa* may delay the events that occur until germination, especially the repair and synthesis of DNA, which would be continued later. This is corroborated by the absence of cytotoxic effects in the longer exposure periods.

It should be noted that for seeds of accession GF004 of bean, lower MI values were already observed in intermediate concentrations of MMS, differently from rice. At the highest concentration, of 15 mg/L, the period of 8 h was sufficient to reduce the MI, when compared to the MMS 15 mg/L group for 4 h. It may be suggested that for bean seeds the process of DNA synthesis and repair may occur in the initial stages of imbibition. Comparing the two bean accessions, the initial germination of the seeds of GF007 was 99%, well above that of GF004, which was 56%. This may have had an influence on the greater sensitivity of the seeds of accession GF004 to the deleterious effects of MMS.

Mutagenicity can also be analyzed by cytogenetic tests in meristematic cells of plant roots by using the frequencies of chromosome aberrations and MNs (Leme and Marin-Morales, 2009). MMS occasioned mutagenic effects by the significant increase in the frequency of chromosome aberrations in meristematic cells of *O. sativa* roots at the concentration of 10 mg/L when exposed for 24 h in accession BGA012099 “Ferrinho” (Table 1) and for 8 and 24 h in accession BGA008070 “Primavera” (Table 2), when compared with the control. In the latter accession, there was a significant increase in chromosome fragments for an exposure period of 24 h in relation to the control group and distilled water. The significant increase in chromosome fragments induced by MMS in meristematic cells of *O. sativa* roots corroborated what has been reported on the clastogenic action of MMS (Kaina, 2004). The capacity of MMS to produce breaks seems to depend largely on generating intermediaries of base excision repair (BER), since studies suggest that the intermediaries of BER generated by the removal of N-methylpurines are toxic and clastogenic (Wyatt and Pittman, 2006).

In accession GF004 of *P. vulgaris*, MMS at the concentration of 10 mg/L for 8 h induced an increase in the occurrence of chromosome lagging in meristematic root cells of accession GF004, which differed statistically from the control, from the distilled water, and from the MMS 10 mg/L for 4 h group. There was also an increase in the total frequency of chromosome aberrations in the meristematic root cells for the MMS 10 mg/L for 8 h group, when compared with the control and distilled water. Similar effects were found for MMS at the concentration of 15 mg/L. In other words, there was an increase in the presence of chromosome lagging and total frequency of chromosome aberrations after exposure for 4 h in relation to all the groups tested (Table 3). The fact that no significant increase in chromosome lagging and chromosome aberrations was seen in periods later than those observed, at concentrations of 10 and 15 mg/L in accession GF004 of bean (p ≥ 0.05), may be explained as being due to mitotic inhibition for an exposure period of 24 h, because the chromosome aberrations evaluated in this study require cells that are undergoing division to be noticed (Rank, 2003). MMS is a clastogenic agent, and thus promotes chromosome breaks and the induction of chromosome lagging in plants, as previously reported in *Capsicum annum* L. (Gulfishan *et al.*, 2011; 2012) and *Vicia faba* L. (Sharma *et al.*, 2009) and also observed in the present study. In accession GF007, MMS did not produce a significant increase in the frequency of chromosome aberrations in the meristematic root cells, when compared with the control (Table 4).

Despite the significant increase in the frequency of chromosome aberrations in accessions BGA012099 “Ferrinho” and BGA008070 “Primavera” of *O. sativa* and in GF004 of *P. vulgaris*, MMS did not induce a significant increase in MN frequency in the meristematic root cells in the two tested accessions of *O. sativa* and *P. vulgaris*, when compared with the control. Furthermore, all MNs found were very small, suggesting that these came from a clastogenic effect. Chromosome aberrations are the main mechanisms in MN formation, but they may not produce MNs in daughter cells, because chromosome lagging, chromosome bridges and chromosome fragments originating from bridges can be corrected, or have their effects minimized (Rao *et al.*, 2008).

### Comet test

**Figure 1:**
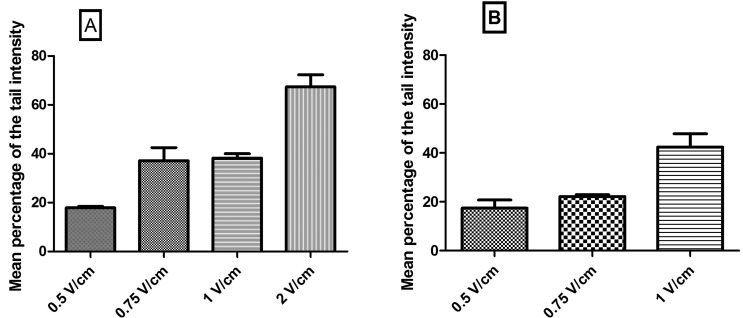
Mean percentage of tail intensity of nucleoids submitted to different voltages in the electrophoresis test in single cell gel. **A**) Embryo nucleoids from *O. sativa* seeds. **B**) Embryo nucleoids of *P. vulgaris* seeds. The data are represented as means ± SEM.

Before carrying out the comet test, different voltages were checked for the electrophoresis stage. This was carried out with the purpose of determining which voltage was most appropriate for observing embryo nucleoids of *O. sativa* and of *P. vulgaris*. As described in the literature, in the control the percentage of DNA found in the tail of nucleoids should be between 10 and 20% to avoid false positive results and statistical errors (Lovell and Omori, 2008). Only the voltage of 0.5 V/cm showed results between 10 and 20%, and it was thus the most appropriate voltage (Figure 1). It should be emphasized that the neutral version of the test was used, in which the nucleoids of cells untreated with a genotoxic agent possess more DNA in the tail region of the comet when compared to nucleoids in the alkaline version of the test (Olive and Banáth, 2006). Therefore, the values of mean intensity from the nucleoid tails in the control experiment for the two *O. sativa* accessions were close to the maximal limit established, which is 20%.

**Figure 2:**
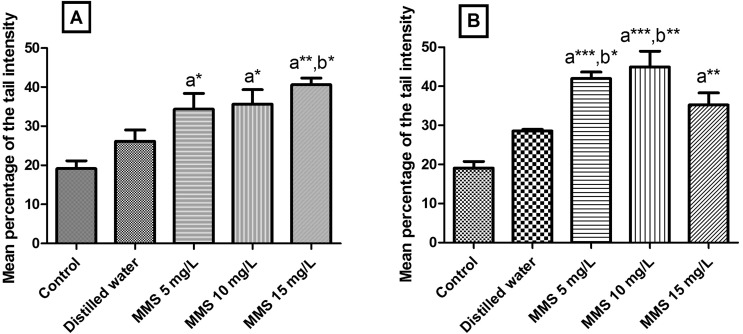
Mean percentage of tail intensity of nucleoids from *Oryza sativa* L. exposed to different concentrations of methyl methanesulfonate (MMS). **A**) accession BGA012099 “Ferrinho”. **B**) accession BGA008070 “Primavera”. The data are represented as means ± SEM. Control = seeds not exposed to MMS or to distilled water. ^a^ Significant in relation to control. ^b^ Significant in relation to distilled water group. One-way ANOVA followed by Tukey test (*p<0.05; ** *p*<0.01; *** *p*<0.001).

**Figure 3:**
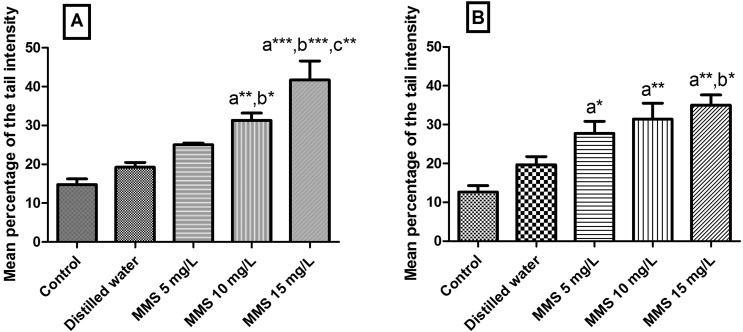
Mean percentage of tail intensity of nucleoids from *Phaseolus vulgaris* L. exposed to different concentrations of methyl methanesulfonate (MMS). **A**) accession GF004. **B**) accession GF007. The data are represented as means ± SEM. Control = seeds not exposed to MMS or to distilled water. ^a^ Significant in relation to control. ^b^ Significant in relation to distilled water group. ^c^ Significant in relation to MMS 5 mg/L group. One-way ANOVA followed by Tukey test (*p<0.05; ** *p*<0.01; *** *p*<0.001).

The results of the comet tests are represented in Figures 2 and 3. MMS led to a significant increase in the tail intensity in the embryo nucleoids of the two accessions of *O. sativa* evaluated at all the tested concentrations, when compared to the control (Figure 2). In the rice accession BGA012099 “Ferrinho”, MMS at the concentration of 15 mg/L also differed statistically from the distilled water group (Figure 2A) and in rice accession BGA008070 “Primavera” the MMS concentrations that differed statistically from the distilled water group were 5 and 10 mg/L (Figure 2B).

**Figure 4:**
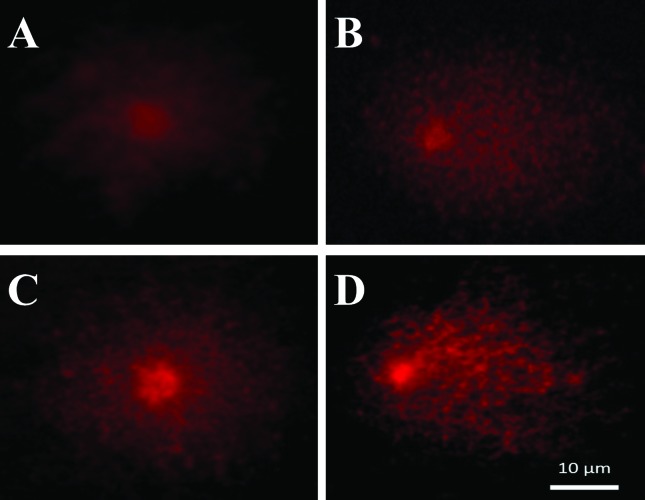
Profiles of nucleoids observed in the comet assay in rice and common beans. **A** and **C**) Nucleoids of *O. sativa* and *P. vulgaris* control embryos, respectively. **B** and **D**) nucleoids of *O. sativa* and *P. vulgaris* embryos exposed to MMS, respectively.

In the accessions of *P. vulgaris*, MMS also produced a significant increase in tail intensity of embryo nucleoids at all the tested concentrations (Figure 3), except at the concentration of 5 mg/L in the accession GF004 of *P. vulgaris* (Figure 3A). In bean accession GF004, there was an increase in nucleoid tail intensity in the concentrations of 10 and 15 mg/L of MMS, when compared to the distilled water group; the concentration of 15 mg/L also differed statistically from the group exposed to MMS at the concentration of 5 mg/L, indicating a possible relationship between the concentration of MMS and the genotoxic effects observed in the comet test. In bean accession GF007, only the concentration of 15 mg/L of MMS differed statistically from the distilled water group (Figure 3B). The profiles of nucleoids obtained in our laboratory for rice and common bean are shown in Figure 4. MMS, when used as positive control to evaluate two auxinic herbicides, at a concentration of 10 ppm, demonstrated high genotoxic activity in a mild alkaline comet test (pH 12.3) in roots of *P. vulgaris* (Cenkci *et al.*, 2010).

The neutral version of the comet test can detect breaks in single and double strands (Collins *et al.*, 2008). Breaks in single strands seen during cell treatment with MMS are probable intermediaries of BER, while breaks in double strands are the result of replication forks that encounter methyl damage or strand breakage produced by intermediaries of BER (Wyatt and Pittman, 2006). However, damage observed during the comet test can be repaired by cell repair mechanisms (Villela *et al.*, 2003). Therefore, the genotoxic damage occasioned by MMS in the two evaluated accessions of *O. sativa* and *P. vulgaris* may be reparable.

The comet test is a very sensitive bioassay that evaluates damage to DNA, and this sensitivity may have contributed to detecting the damage occasioned by MMS at concentrations that did not produce significant effects in the cytogenetic tests. In addition, this test was carried out soon after exposing rice and bean seeds to MMS, but the seeds needed to have germinated for the cytogenetic tests after exposure to MMS. Germination and growth took 2 to 5 days for roots to be obtained at the ideal size for cytogenetic tests. This period may have been sufficient to repair damage to DNA (Villela *et al.*, 2003). It is worth highlighting the value of cytogenetic tests together with the comet test, as together they provide a better understanding of the results obtained, because damage such as MN formation, is irreversible in cells, whereas damage seen in the comet test can be repaired (Vasquez, 2010).

## Conclusion

Rice and beans showed sensitivity to the cytotoxic, mutagenic and genotoxic effects of MMS. This study provides contributions to the standardization of methodologies to evaluate cytotoxicity, mutagenicity and genotoxicity in rice and beans. It was shown that the comet test is more sensitive than the cytogenetic tests used. The comet test, in particular, can be proposed for the measurement of genomic instability in accessions of rice and beans in gene banks. This may contribute to establishing sensitive tools to detect deterioration caused by accelerated aging and storage conditions in gene banks.
